# Production of Exopolysaccharide-Based Porous Structures for Biomedical Applications: A Review

**DOI:** 10.3390/nano13222920

**Published:** 2023-11-09

**Authors:** Alessandra Zanotti, Lucia Baldino, Ernesto Reverchon

**Affiliations:** Departement of Industrial Engineering, University of Salerno, Via Giovanni Paolo II, 132, 84084 Fisciano, Italy; azanotti@unisa.it (A.Z.); ereverchon@unisa.it (E.R.)

**Keywords:** exopolysaccharide, drying, supercritical carbon dioxide, scaffold, tissue engineering

## Abstract

Exopolysaccharides, obtained from microorganisms as fermentation products, are interesting candidates for biomedical applications as scaffolds: they are biocompatible, nontoxic, antimicrobial, antitumor materials. To produce exopolysaccharide-based scaffolds, sol–gel technology could be used, which ends with the removal of the liquid phase from the polymeric network (i.e., the drying step). The aim of this review is to point out the most relevant strengths and weaknesses of the different drying techniques, focusing attention on the production of exopolysaccharide-based porous structures. Among these drying processes, supercritical carbon dioxide-assisted drying is the most promising strategy to obtain dried gels to use in the biomedical field: it produces highly porous and lightweight devices with outstanding surface areas and regular microstructure and nanostructure (i.e., aerogels). As a result of the analysis carried out in the present work, it emerged that supercritical technologies should be further explored and applied to the production of exopolysaccharide-based nanostructured scaffolds. Moving research towards this direction, exopolysaccharide utilization could be intensified and extended to the production of high added-value devices.

## 1. Introduction

Exopolysaccharides (EPSs) are an interesting class of carbohydrate biopolymers, naturally secreted through a defense mechanism by microorganisms (e.g., bacteria, fungi, algae) in an extracellular manner [1,2,3]. EPS production is associated with nutrient and moisture regulation in microbes’ environment and with protection against desiccation and toxic substances [1,4]; moreover, they are crucial for microbe–host interactions [5].

Because EPSs are produced as a response to variations [6] in the microbial environment [7] such as temperature, pressure, salinity, pH, etc., their industrial production relies on the optimization of fermentation techniques and their relative parameters (e.g., culture temperature, culture period, carbon concentration, and C/N ratio [8,9]). Indeed, these polysaccharide biopolymers are easily recovered and separated from the fermentation broth (i.e., the growth medium) and centrifugated to obtain the highly viscous exopolysaccharide product [6,10]. Moreover, both the starting microorganism and fermentation parameters can affect the structural chemistry of the EPS produced.

**Figure 1:**
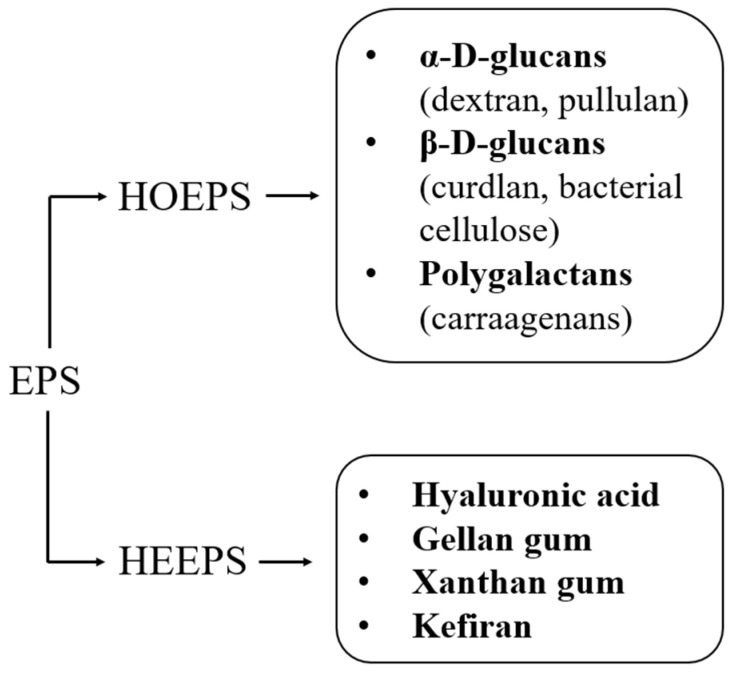
Classification of the most widespread EPSs.

From a chemical point of view, EPSs can fall into two main categories: homoexopolysaccharides (HOEPSs) and heteroexopolysaccharides (HEEPSs). The first category is characterized by polysaccharides whose structural backbone is obtained from the repetition of a single monosaccharide unit, whereas the second category contains polysaccharide biopolymers obtained by the concatenation of different types of monosaccharides [6,11]. Within the category of HOEPSs, there are α-D-glucans, β-D-glucans, fructans, and poly-galactans [1]. On the other hand, HEEPSs are a wider category of exopolysaccharides since there can be multiple combinations of different monomeric units (e.g., glucose, fructose, galactose, sorbitol, mannose [12]): examples of HEEPSs are hyaluronic acid, gellan gum, xanthan gum, and kefiran. A brief schematic representation of the most abundant EPSs is shown in Figure 1.

EPSs show desirable properties that make them eligible for pharmaceutical and biomedical applications: they are hygroscopic, biocompatible, non-cytotoxic, and have antimicrobial, anti-inflammatory, and antitumor properties [13,14,15].

With a view to the biomedical field—in particular, to tissue engineering (TE) and regenerative medicine—3D-nanostructured and porous materials and their composites are crucial. Indeed, scaffolds play a pivotal role in TE, as a supporting structure to implanted cells, to favor their growth, differentiation, and vascularization; moreover, scaffolds should biodegrade in time to make space for the new tissue [16,17]. Therefore, scaffolds should meet some specifics, such as very high porosity (to allow nutrients exchange and the release of metabolic wastes), suitable pore size distribution (according to the tissue being replaced), enhanced nanostructure (to mimic the extracellular matrix (ECM) and to promote cell proliferation, migration, and differentiation), hierarchical morphology (on the macroscale, mesoscale, and nanoscale), pore interconnectivity, biocompatibility, programmable biodegradability, lack of toxicity, highly specific areas, mechanical resistance, etc. [18,19,20,21,22,23,24]. Polymers, both organic and inorganic, are employed in TE; nevertheless, natural materials meet most of the specifics listed previously (i.e., biocompatibility, biodegradability, nontoxicity).

**Figure 2:**
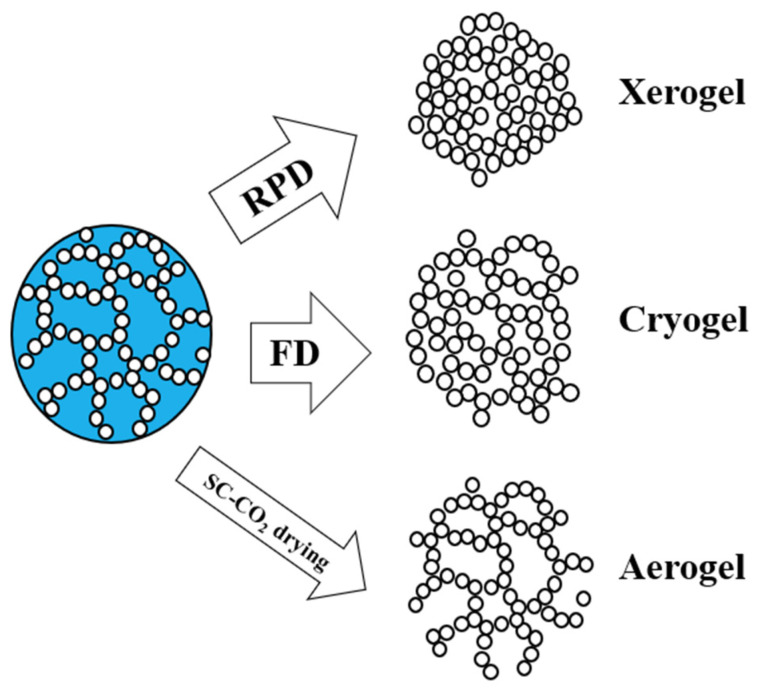
Gel drying routes and final products.

To produce scaffolds, a possible strategy is the preparation of the biopolymeric solution and its subsequent gelling to obtain a hydrogel or a solvogel. Then, the drying step (i.e., the removal of the solvent entrapped in the polymeric network) influences the final properties of the scaffold. Several routes might be followed to dry a polymeric gel, namely, room pressure drying (RPD) to form a xerogel, freeze-frying (FD) to produce a cryogel, and supercritical CO_2_ (SC-CO_2_) drying to produce an aerogel [25]. However, due to high stresses exerted on polymeric chains by the liquid phase, both xerogels and cryogels are characterized by an overall collapsed structure. Consequently, irregular macropores are observed and low specific surface areas are obtained since the nanostructure is sacrificed: the original nanoporosity of the hydrogel or solvogel is compromised [25]. On the other hand, SC-CO_2_ drying allows the regular hierarchy of the starting gel on every scale to be preserved [26] since SC-CO_2_ has negligible surface tension and gas-like diffusivity. A schematic representation of the different drying routes and their final product is depicted in Figure 2.

Aerogels are the most interesting candidates for TE applications since they can mimic the human tissues on different scales; moreover, they are lightweight, porous, low-density materials with outstanding surface areas [27]. Natural polymers are favorable for aerogel production, especially for TE applications [28]. EPSs are eligible to produce 3D scaffolds, although they do not possess some characteristics needed for these applications (e.g., tensile and compressive strength); however, their composites can overcome this issue [29].

The combination of EPSs’ properties with 3D porous materials could be a powerful tool for high-end applications. Nevertheless, the technology underlying the production of such devices is crucial to their final performance. For this reason, the aim of this work is to sum up and critically review the most relevant findings about the production of EPS-based 3D porous materials for biomedical applications. Attention was mainly focused on published research in the timespan of 2014–2023. This line of work should highlight the most important features of several EPSs (both HOEPSs and HEEPSs) and their production techniques, moving research towards the full valorization of these valuable biomaterials.

## 2. EPS-Based Porous Devices Production

### 2.1. Homoexopolysaccharides

#### 2.1.1. Alpha-D-Glucans

α-D-glucans are EPSs whose monomeric unit is D-glucose. As mentioned previously, there could be different types of α-D-glucans depending on the position of chain links. There could be: (i) dextran, in which a linear structure is obtained by (1→6)-linked α-D-glucose units, and few branches might be present due to (1→2), (1→3), and (1→4)-linked glycosidic units; (ii) mutan, mainly characterized by linear (1→3)-linked α-D-glucose units; (iii) alternan, made up of glycosidic units linked by alternating (1→3) and (1→6) glycosidic bonds; (iv) reuteran, whose main glycosidic link is (1→4) [30,31]; (v) pullulan, consisting of maltotriose units (three glucose units linked by α-1,4 glycosidic bonds) linked by α-1,6 glycosidic bonds [32]. Among this list of α-D-glucans, most papers are focused on dextran (DEX) and pullulan (PUL).

**Figure 3:**
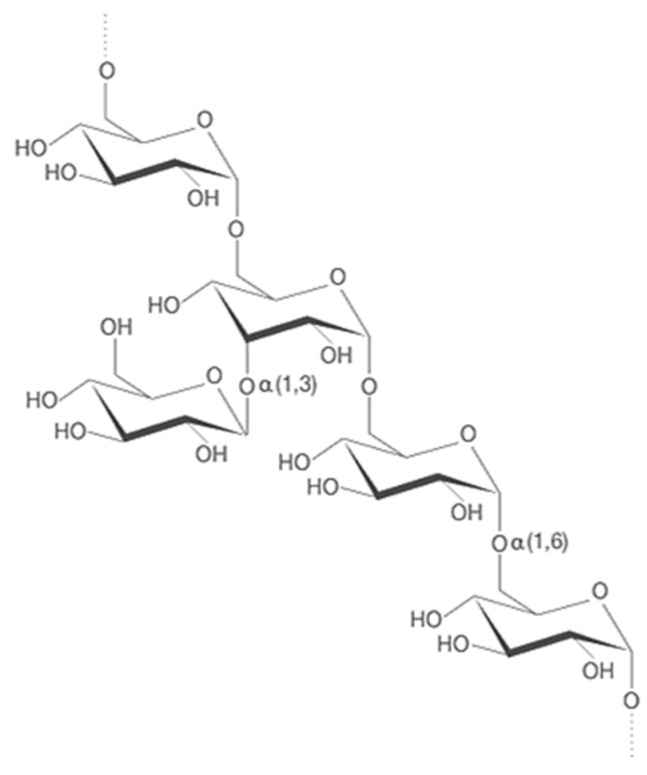
Dextran chemical backbone.

Dextran can be obtained using microorganisms like lactic acid bacteria (LAB), such as *Leuconostoc*, *Weissella*, *Lactobacillus*, and *Streptococcus* [33,34]. Its structure is reported in Figure 3.

Depending on how the synthesis is carried out, dextran could have a variable degree of branching or molecular weight [35]. It has an extendable coil structure in solution, and it is soluble in water and several organic solvents (e.g., dimethylsulphoxide, ethylene glycol, glycerol) because of (1→6)-glycosidic linkages [34,35,36,37]. Some bacterial strains (i.e., *W. confuse* H2, *W. confusa* VP30, *Leu. Pseudomesenteroides* DRP-5) can produce dextran-based EPSs with high molecular weight (around 10^6^ Da), although other strains might release EPSs with different molecular weight (from 10^4^ to 10^8^ Da) [38]. From an engineering perspective, EPS molecular weight affects the gelation process prior to the production of scaffolds: the lower the molecular weight, the fewer the entanglements between polymeric chains and the less intense the cohesive forces between them. On the other hand, higher molecular weights could result in a close and compact gel structure, which might not be favorable for scaffold production.

The dextran gelation process is a topic discussed in research: being overall neutral, DEX forms a thermoreversible gel in water due to hydrophilic interactions, whereas, if cations are present in water, positive charges displace the polymeric chain–chain interactions and the gelling process is compromised [39]. Also, concentration affects the polymeric chains’ disposition in solution: namely, at lower concentrations, dextran shows a random coil geometry; in contrast, at higher concentrations, chains adopt a compact coil geometry [40].

Some attempts to produce dextran-based scaffolds are reported in the literature. Nikpour et al. [41] added bioactive glass ceramic (BCG) to DEX to produce nanocomposite scaffolds. To improve the cross-linking degree, epichlorohydrin (ECH) was added to the polymeric solution; then, to ensure a complete cross-linking reaction, the hydrogel was heated up to 60 °C for 24 h. The resulting product was, at last, freeze-dried at −48 °C. SEM images showed that the resulting cryogel had irregular macropores; in addition, mechanical tests highlighted that BCG content affected compressive modulus. Indeed, by increasing the particle content in DEX gel, mechanical properties improved (compressive modulus increased from 1.3 kPa to 76.61 kPa after the addition of 2% *w*/*w* of BCG); although, for higher contents (from 4 to 16% *w*/*w*), the trend reversed, probably due to an ineffective dispersion in the polymeric network. Also, Ghaffari et al. [42] used ECH as a cross-linker to produce dextran/nanocrystalline β-tricalcium phosphate (β-TCP) nanocomposite scaffold by freeze-drying. In this work, it was highlighted, by FT-IR analysis, that traces of ECH were present in the final product: being ECH toxic and carcinogenic, its presence in scaffolds compromises their application in the biomedical field. Also, in this case, FD produced macropores of about 100 μm.

Increasing additive concentration, β-TCP agglomerated to form a cluster-like morphology. Therefore, this drying technique might not ensure a completely homogeneous distribution of nanoparticles in DEX-based scaffolds. El-Meliegy et al. [43] obtained dextran–chitosan composite scaffolds by freeze-drying, with the addition of nano-hydroxyapatite, to mimic bone tissues. These authors did not use cross-linkers to promote the formation of a stable gel: this procedure is important in TE applications in which less-toxic substances are used to obtain more appealing and safe final products. Moreover, previous considerations on the effect of nanoparticle addition to the scaffold also apply in this case. Indeed, compressive strength increased (0.18, 0.58, 0.59, 0.63 MPa) with the content of nano-hydroxyapatite (0, 20, 30 and 40%), but the trend was reversed for pore diameter (66.7, 56.6, 53.1, and 47.4 μm), meaning that nanoadditives agglomerated in scaffolds’ pores and reduced the overall pore volume. Even though the trends of mechanical properties were satisfying, these values were far from being eligible for bone regeneration. These results could be related to the absence of a proper nanostructure that may host homogeneously nano-hydroxyapatite crystallites and enhance mechanical properties. Pacelli et al. [44] also proved that dextran-polyethylene glycol cryogels are not cytotoxic.

**Figure 4:**
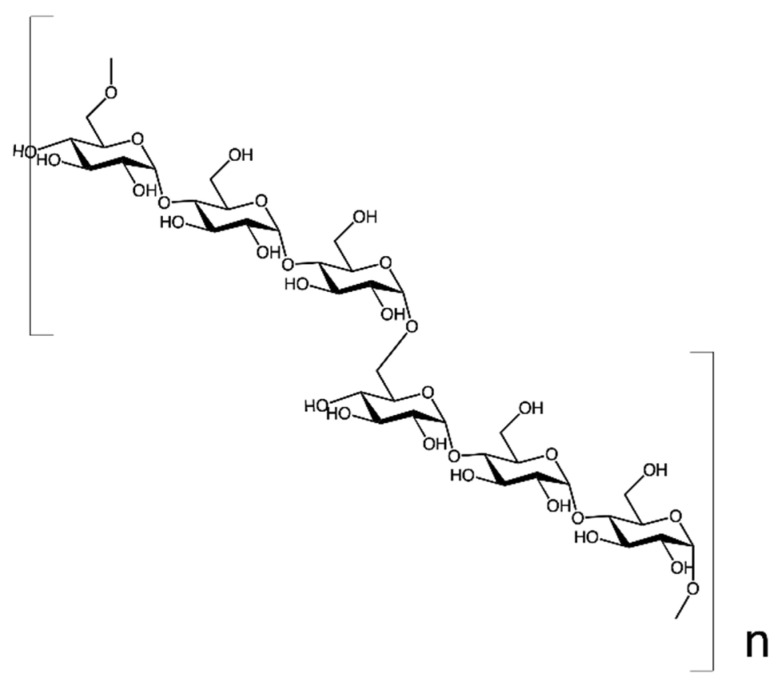
Pullulan structure.

Pullulan is a microbial EPS that consists of α-l,6-linked maltotriose units obtained from fungi (e.g., *Aureobasidium pullulan*) [45,46]. In Figure 4, the chemical structure of PUL is shown.

Being a linear polymer with no branching, PUL is more soluble in water than dextran (which is relatively more branched); in aqueous solution, PUL forms random coils [47]. Its biomedical applications are related to its biodegradability, antioxidant properties, and biocompatibility; however, its poor mechanical properties, pH sensitivity, and antimicrobial properties could be improved by physicochemical modifications (e.g., esterification, etherification, periodate oxidation, etc.) [45].

Yang et al. [48] produced gelatin/pullulan porous structures by freeze-drying the polymeric solution for 3 days followed by a 5-day-long Maillard reaction in a chamber kept at 70 °C. After this week-long production step, the obtained structures were characterized. SEM images outlined that a macroporous structure was obtained after FD, and that Maillard reaction partly compromised structure regularity: therefore, these biopolymers might not be stable at high processing temperatures. Moreover, it is noteworthy that PUL scaffolds did not show round pores—they were more elongated: pores evolved to a round-like shape when gelatin concentration in solution increased. This result could be related to pullulan behavior in solution and its gelation phenomena, apart from ice crystals nucleation, growth, and sublimation. Moreover, mechanical strength was studied: PUL alone showed a plastic behavior (and a compressive strength of 0.34 MPa, measured at 20% strain), and the addition of gelatin widened the elastic region. Compression tests highlighted an interesting behavior: compressive strength increased (although not significantly) after the cross-linking reaction, but as far as morphology was concerned, denser structures were obtained. In this case, a Maillard reaction might be unnecessary for the final application since it is also partly detrimental to scaffolds’ morphology.

On the other hand, Zhao et al. [49] performed a silanation process on a PUL gel (blended with polyvinyl alcohol (PVA)), to obtain enhanced performance in terms of mechanical resistance and hydrophobicity; then, it was supercritically dried and characterized. Compression modulus increased from 4 kPa to 234 kPa, and compressive strength at 50% strain increased from 8 kPa to 164 kPa, for PUL/PVA aerogels and silanated PUL aerogels, respectively. It is noteworthy that SEM images showed an overall nanoporous structure, and the pores’ average diameter was 60 nm; this result outweighs the ones related to freeze-dried gels, whose pore diameters were around 100 μm [48]. In contrast, mechanical properties worsen when moving from FD to SC-CO_2_-assisted drying (0.34 MPa to 8 kPa), likely due to aerogels’ higher air volume. The nanoporosity of SC-CO_2_-dried PUL-based gels was also confirmed by specific surface area (SSA) measurements: these aerogels demonstrated an SSA of 617 m^2^/g. Supercritically dried gels also showed low densities (around 0.099 g/cm^3^) according to the data in the literature on aerogel bulk characteristics [50]. Indeed, the comparison among these works highlights that SC-CO_2_-assisted drying results in the preservation of the nanostructure, and bulk properties are positively affected, even though mechanical properties should be improved.

#### 2.1.2. Beta-D-Glucans

β-D-glucans differ from α-D-glucans because of the anomeric conformation of glucose [51]. The abundance of hydroxyl groups on their structural backbone allows these EPSs, in their original or modified configuration, to hold water molecules [52]; thus, bioavailability and biocompatibility are enhanced. On the other hand, β-D-glucans can arrange themselves in various configurations (i.e., random coil; single, double, or triple helix; aggregates, etc.), based on the solubilization medium [53]. Hence, polymeric conformation might affect the final bioactivity of the material; however, this issue still needs to be fully addressed. Some of the most discussed β-D-glucans are curdlan and bacterial cellulose.

**Figure 5:**
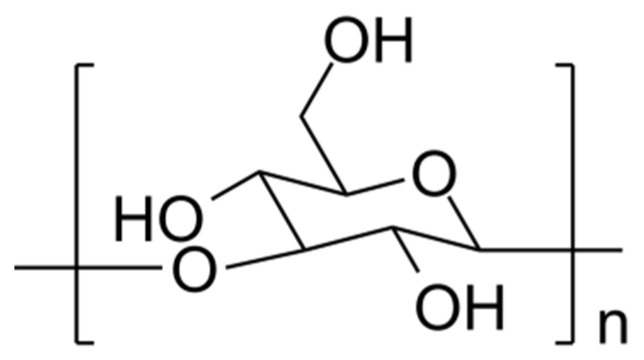
Curdlan monomeric unit.

Curdlan (CUR, named after its ability to curdle when heated) is a linear EPS, as shown in Figure 5 (obtained from *Alcaligenes faecalis* and *Agrobacterium* strains) [46]. CUR is insoluble in water because of intense intra/intermolecular hydrogen bonds, but it can be solubilized in alkaline solutions [54]. However, curdlan behaves differently depending on the solution temperature: gelation occurs in different ways. For instance, when a CUR aqueous solution temperature is set between 80 and 130 °C, a high-set thermal nonreversible resilient gel is formed; whereas, when temperature is set at 55 °C, a low-set reversible gel is formed. High-set gels are formed mainly by triple helixes and low-set gels by single helixes [55,56]. Nevertheless, the phenomena underlying the gelation process are still to be completely understood.

**Figure 6:**
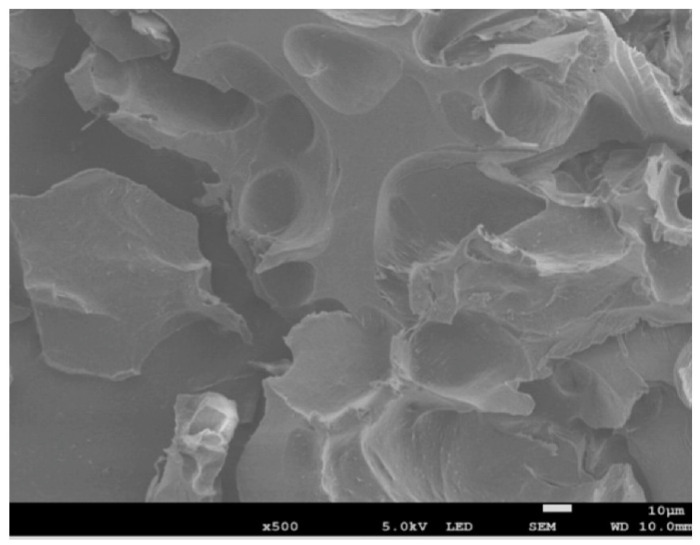
SEM image of CUR/whey protein cryogel (adapted from [58]).

Because curdlan is nontoxic and biodegradable, it is used in regenerative medicine (e.g., for biocompatible bone scaffolds). Some attempts to produce curdlan-based scaffolds are reported in the literature. Przekora et al. [57] produced CUR xerogels by room pressure drying to test their biodegradability and their ability to host osteoclasts and promote their proliferation, with a view to bone TE. These authors prepared a high-set hydrogel, heating the aqueous CUR solution up to 90 °C. SEM images of curdlan-processed samples highlight an overall closed and smooth surface; however, the focus was on CUR xerogels to host and promote osteoclasts proliferation. Indeed, the results showed that CUR represents a hospitable environment for these cells. However, to use CUR-based scaffolds for regenerative medicine, it is imperative to improve these materials’ characteristics. Klimek et al. [58] freeze-dried CUR (M_w_ = 80 kDa, concentration about 8% *w*/*w*)/whey protein composites to assess their cytocompatibility towards human chondrocyte, morphology, and mechanical behavior. SEM observations outlined that CUR composite scaffolds possessed a macroporous morphology (pore diameter about 10 μm), as can be seen in Figure 6.

Moreover, compression tests showed that this composite had a Young’s modulus of 0.849 ± 0.157 MPa. According to the authors of this work, this value was acceptable for scaffolds intended for cartilage regeneration. Also, in this case, a CUR-based scaffold promoted cell proliferation and differentiation, and the scaffold degraded in a collagenase-rich environment in about 9 weeks. El-Naggar et al. [59] adjusted CUR cryogels’ brittleness and fragility by adding polyethylene oxide to the polymeric solution and glyoxal as cross-linker. They pointed out that cryogels’ cross-section was macroporous and lamellar, resembling ice crystals grown within the polymeric structure. In addition, even though the Young’s modulus increased with the polyethylene oxide content, values were still not comparable to the ones produced by Klimek and coworkers [58] since the maximum value reached was 0.40 MPa. Indeed, to improve CUR gels’ macroscopical behavior, attention must be paid to the interactions between the materials when the filler is selected. Furthermore, glyoxal (used as a cross-linker between the polymeric phases) is toxic and irritating to human skin: its utilization should be controlled or, at least, it should be removed completely from the obtained product. To the best of our knowledge, there are still no attempts to produce CUR-based aerogels by supercritical drying: indeed, this route is worth following due to curdlans’ interesting characteristics and the possibility of extracting toxic cross-linkers while drying [60].

**Figure 7:**
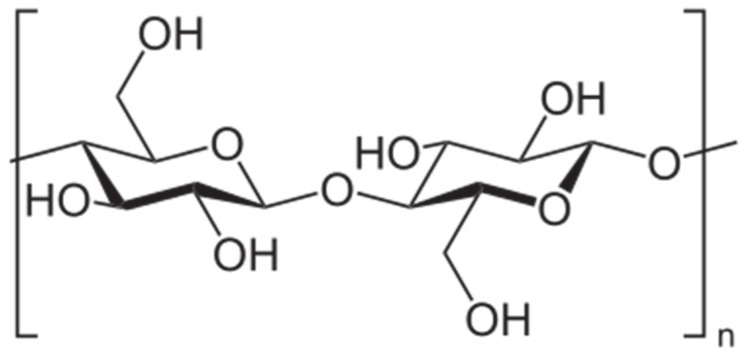
Cellulose monomeric unit.

Bacterial cellulose (BC) is a kind of cellulose produced extracellularly by some microorganisms (e.g., *Acetobacter xylinum*, *Sarcina ventriculi*, *Pseudomonas*, etc. [61]). In general, cellulose is one of the most abundant biopolymers on earth. Therefore, to date, lots of studies have been carried out on the matter. The growing interest in cellulose stems from its complex morphological hierarchical organization. Indeed, it may possess bundles or aggregates of superfine fibrils which contain both amorphous and crystalline domains [62]. BC is an EPS known for being resilient, biomimetic, and biocompatible; thus, it is eligible for biomedical application as scaffolds to repair nerves, skeleton, and hamstring [63]. Its chemical structure is reported in Figure 7.

For example, Huang et al. [64] produced porous BC scaffolds (from *A. xylinum X-2 strains*) by FD, blending BC with agarose. Hydrogels were freeze-dried at −50 °C for 2 days; moreover, they studied the effect of cross-linking by procyanidins and of the addition of gelatin and hydroxyapatite particles. Even though the pores’ dimension fell into the range 20–300 μm, each kind of scaffold promoted cell viability, proliferation, and osteogenic differentiation. Expectedly, mechanical properties improved after blending with additives and cross-linking. Nevertheless, to completely remove water from the macrostructure, 2 days were needed. Although BC and its composites showed favorable characteristics for TE applications, its properties could be better exploited, and process time could be shortened. Following this line of thought, Ganesan and coworkers [65] compared different drying techniques to obtain cellulose porous structures. Even though this paper does not focus on cellulose produced by bacteria, the considerations that they pointed out are appliable also to BC. To produce hydrogels, cellulose was solubilized in water and calcium thiocyanate tetrahydrate kept at 117 °C. Then, after 16 h, the hydrogel was washed using isopropanol. Some samples were put in oven at 50 °C for 3 days to produce xerogels. On the other hand, to produce cryogels, hydrogels were freeze-dried at −50 °C for 48 h. These procedures totaled an amount of at least 3 days to produce cryogels or xerogels. Moreover, aerogels were produced by supercritical drying. The comparison among these drying techniques is representative of their effectiveness, according to the considerations stated in the Introduction. Indeed, supercritical drying led to the lowest volume shrinkage (i.e., about 15%), whereas FD and RPD led to dramatic collapses of the structure (40% and 90%, respectively). Cellulose aerogels showed SSA values of up to two orders of magnitude greater than differently dried gels (e.g., 300 m^2^/g vs. 23 m^2^/g of cryogels and 0.81 m^2^/g of xerogels). SEM images proved that cellulose aerogels were characterized by a fiber-like structure on both the microscale and nanoscale; in addition, pores were open, and chains were interconnected. Freeze-dried gels were only macroporous, whereas xerogels’ (obtained in ethanolic solution) cross-section was almost completely closed. Therefore, from a morphological point of view, aerogels can be considered superior over differently dried gels. However, mechanical properties are favorable to xerogels rather than aerogels or cryogels, even though the Young’s modulus values fall in an order of magnitude of mega-Pascal for every product. This result could be associated with the extreme compactness of xerogels, whereas cryogels and aerogels are far more porous, and their mechanical properties might be affected by a higher air volume fraction. For this reason, other authors explored the effects of supercritical drying on the production of BC-based aerogels [66,67,68]. They all agree that these materials are outstanding in terms of hierarchical and regular morphology, SSA, and biomechanical performances.

#### 2.1.3. Polygalactans

**Figure 8:**
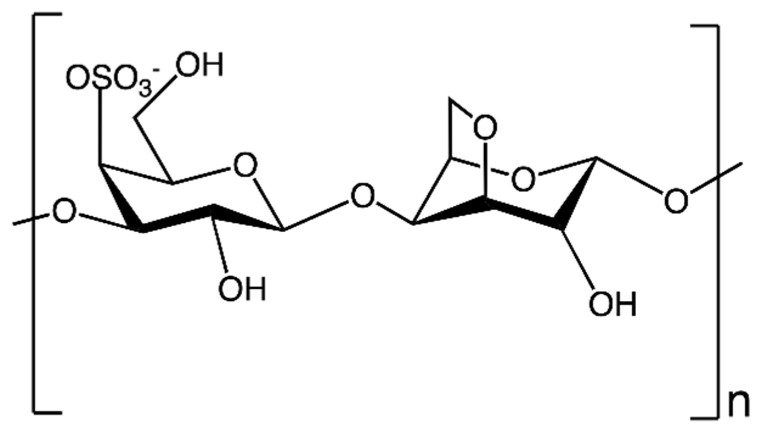
Monomeric unit of κ-carraagenan.

Polygalactans are biopolymers obtained by a repeating unit of galactose [69]. They can be obtained from both algae and some strains of bacteria [46,70]. In particular, the most interesting polygalactans, to the best of our knowledge, are carrageenans (CARs). CARs are linear sulfated polysaccharides formed by (1→4)-linked β-D-galactose and anhydrogalactose units. These EPSs are obtained mostly from red seaweed species (e.g., *Chondrus crispus*, *Eucheuma cottonii*, *Gigartina stellate*, *Kappaphycus alvarezii*) [46,70,71,72]. In the literature, at least 15 types of CARs are reported, which differ from one another by the abundance of sulphate groups, presence of anhydrogalactose, chemical structure, etc. [71]. Among this wide range of CARs, the CAR most employed for biomedical purposes is κ-carrageenan (κ-CAR), which shows an ester sulphate content between 25 and 30% and anhydrogalactose relative amount between 28 and 35% [73]. Indeed, κ-CAR is interesting for its antiviral, antibacterial, antihyperlipidemic, anticoagulant, antithrombotic, antitumor, and immunomodulatory properties. Therefore, this biopolymer is versatile and may be used in several biomedical applications like cartilage and bone tissue engineering. Its similarity and affinity to glycosaminoglycans (i.e., chitosan) and ability to form polyanionic complexes [70,74] also contribute to its versatility. Its monomeric unit is depicted in Figure 8.

κ-CAR gelation behavior is a debated topic of research: indeed, it can form thermoreversible gels in aqueous solutions upon cooling, but the presence of different cations might affect gel conformation [75]. Potassium cations induce the formation of a double helix structure, disentangling κ-CAR coils, whereas sodium cations do not promote the formation of a strong gel but of a disordered and weak one [75,76]. Also, divalent cations (such as Ca^2+^) seem to affect gelation behavior [74], while polymer concentration and process temperature influence its kinetics [77].

To begin with, Loukelis et al. [78] produced κ-CAR/chitosan/gelatin scaffolds for bone tissue engineering by FD. According to previously discussed results for other EPSs, freeze-dried gels also showed, in this case, a macroporous morphology: pore size distribution fell into the range 100–160 μm, and porosity was estimated to be above 80%. In this work, the crucial role that the cross-linker plays in gel formation and, thus, its stability, was highlighted: for the samples produced using the same biopolymer concentration, the addition of potassium chloride (KCl) resulted in improved mechanical performances. For example, the scaffold produced by the blend of κ-CAR, chitosan, and gelatin showed a Young’s modulus of about 100 kPa, whereas the same blend, but cross-linked with KCl, presented a Young’s modulus of about 200 kPa, which, therefore, doubled with respect to the non-cross-linked blend. This means, once again, that the addition of a cross-linker results in a more rigid structure and in improved mechanical resistance. Moreover, these authors proved that this biopolymeric blend favors osteogenic differentiation. Also, κ-CAR hybrid nanocomposite cryogels were produced [79]. In this case, cross-linking was carried out using *N*,*N*′-methylene-bis-acrylamide (NN-MBA), and nano-hydroxyapatite and graphene oxide were added to the polymeric solution. It is worth mentioning that the addition of nanoadditives significantly improved the value of the Young’s modulus: it increased up to 442.63 ± 6.3 MPa.

Manzocco and coworkers [80] produced κ-CAR-based aerogels. In order to guarantee a satisfying cross-linking degree, the aqueous solution of κ-carrageenan was poured into a coagulation bath (an aqueous solution of KCl); once the water was replaced by ethanol, the alcoholgel was supercritically dried at 11 ± 1 MPa and 45 °C, using a variable CO_2_ flow rate during the process. Overall, this SC-CO_2_ drying strategy lasted around 9 h. However, sample shrinkage was non-negligible; the same phenomenon was observed during the solvent exchange step. This problem could be related to a non-gradual solvent exchange step due to an initial ethanol concentration in solution that was too high. The supercritical drying step also contributed to sample shrinkage, but with increasing κ-CAR concentration, shrinkage was limited, meaning that the gel networks were stronger. Indeed, gel network strength influences shrinkage phenomena: if intermolecular bonds are not intense enough, once the solvent is removed, polymeric chains tend to turn back to a random coil conformation, reducing overall sample volume [81]. Some authors focused their attention on supercritical techniques [82] to further investigate the morphological properties of κ-CAR-based aerogels. SEM images of these aerogels displayed open and interconnected pores at the microscale, but, even though a microstructure existed, pore mean diameter was below 1 μm, unlike that of freeze-dried gels, whose pores’ mean diameter was generally about 100 μm. In addition, even though SEM images on the nanoscale were not reported, SSA measurements can be a proof of the existence of a nanostructure: SSA values of 2% *w*/*w* κ-CAR aerogels were up to 221 m^2^/g, and pore volume calculated by adsorption isotherms was about 20–50 nm, according to SSA values. Ganesan and Ratke [83], on the other hand, showed not only that potassium thiocyanate (KSCN) can be completely removed, but they also obtained SEM images of these aerogels on the nanoscale (down to 400 nm). SEM images showed that κ-CAR-based aerogels had a homogenous, fiber-like, open, and interconnected morphology even on the nanoscale. These images, coupled with those on the microscale [82], proved that supercritical-assisted drying produced the most regular morphology when compared to other drying techniques.

Overall, in Table 1, a summary of the most important advantages and disadvantages related to the production of some HOEPS-based porous structures is reported.

Some aspects emerge from the synthesis of Table 1:

- Regardless of the HOEPS used, FD leads to macroporous devices, useful for nutrient exchange in TE but unable to mimic human tissues and to host nanoparticles without clustering;
- When working with HOEPSs, it is likely that cross-linkers will be needed to promote the formation of a stable, strong, and resilient polymeric network;
- SC-CO_2_ drying is the only technique that enables complete cross-linker removal during the process, thus improving biocompatibility;
- Aerogels often suffer from poor mechanical properties due to their high air volume: some fillers must be added to improve their characteristics;
- In general, HOEPSs are proven to be biocompatible and eligible for TE.

### 2.2. Heteroexopolysaccharides

HEEPSs, contrarily to HOEPSs, are characterized by a chemical backbone made up of different monomeric units. Their properties strongly depend on the kind of monosaccharide that composes the structure. Although there could be a wide range of HEEPSs, research has focused attention only on some of them, namely:

- Hyaluronic acid (HA), a linear polysaccharide obtainable from *Streptococcus thermophilus* and composed of alternating N-acetylglucosamine and glucuronic acid [84]. This polysaccharide is now used in therapeutics, drug delivery, oncology, vascular tissue, cartilage, bone, and skin tissue engineering [85]. It is advantageous for scaffolding because of its biodegradability, biocompatibility, bioresorbability, abundance in connective tissues, presence of functional groups that enhance reactivity, etc. [86];
- Gellan gum (GG), obtained from *Sphingomonas paucimobilis* and made up of repeating units of monosaccharides, such as glucose, glucuronic acid, glucose, and rhamnose [87]. It is a linear anionic high-molecular-weight exopolysaccharide [88]. It is water-soluble, easy to fabricate, biocompatible, biodegradable, and allows hydrogel formation [89];
- Xanthan gum (XG)—produced by *Xanthomonas*—which consists of D-glucosyl, D-mannosyl, and D-glucuronyl acid residues [90]. XG is appealing due to its nontoxicity, excellent biocompatibility, and immunization properties, apart from its antitumor effects [91];
- Kefiran (KEF), a branched glucogalactan obtained from various *Lactobacillus* strains present in kefir grains [92]. It is interesting for biomedical applications due to its antibacterial, antitumor, anti-inflammatory, and antioxidant properties [93].

Even though these HEEPSs strongly differ from one another because of their chemical backbone, they all have gelation behavior in common. Indeed, the abundance of functional groups allows the listed HEEPSs to form gels under the influence of temperature (upon cooling, the random coil conformation rearranges to more ordered domains [94,95]) or because of physical (e.g., divalent cations [96]) and chemical (e.g., carbodiimides, aldehydes, sulphides, polyfunctional epoxides) cross-linkers [86]. In some cases, gelation behavior and its overall properties are affected by the acetylation degree [86,96]. Due to the appealing properties of HEEPSs, some attempts to produce scaffolds for TE applications are reported in the literature. However, most of them focus on the production of hydrogels and only a few on their drying.

HA-based hydrogels are proven to be effective for vocal fold [97], bone, cartilage [98], and cardiovascular [99] tissue engineering. HA cryogels and their composites have been produced by Suner et al. [100] and Najberg et al. [101]. The former obtained HA hydrogels by cross-linking using divinyl sulfone. Moreover, they added halloysite nanotubes to improve mechanical and thermal behavior as well as cell adhesion, proliferation, and nutrient exchange. Once freeze-dried, the materials were characterized and the authors proved that HA-based scaffolds were worthy of mention in TE applications since they promoted cell viability, proliferation, and adhesion effects. However, their mechanical properties were poor (the maximum value of the Young’s modulus was 99 ± 4 kPa for HA/halloysite 1:2 cryogels). On the other hand, Najberg and coworkers [101] also produced HA-based cryogels for soft tissue engineering applications by freeze-drying. SEM images showed only a macroporous morphology, but mechanical properties and biodegradation were coherent with those of brain tissue. Therefore, it is interesting to point out that HA properties can be tuned using different nanofillers or polymeric blends to mimic the desired tissue. Aguilera-Bulla et al. [102] used SC-CO_2_-assisted drying to produce HA-based cross-linker-free aerogels. However, to ensure the formation of an interconnected structure, they adjusted solution pH to enhance chain–chain interactions. They performed gel drying using a multi-step procedure, moving from 50 bar and 37 °C (non-supercritical conditions) to 80 bar and 37 °C (supercritical conditions) to ensure complete solvent removal. They concluded that HA-based dried structures performed best when the solution was acidic, whereas, when pH increased (i.e., the solution was basic), polymeric chains did not withstand the drying process and collapsed due to weak intermolecular interactions. It is noteworthy that SEM images outlined that the HA nanostructure was intact, and it resembled a fiber-like network. As an ulterior proof of natural aerogel morphological hierarchy, SSA was measured to be 510 m^2^/g.

Gellan hum hydrogels have been produced and tested to mimic soft and hard tissues [103,104,105,106]: this material favored cell adhesion and proliferation. Regarding the drying of GG-based structures, Cassanelli and colleagues [107] studied the effects of the drying technique on the final product. Namely, they oven-dried some gels, freeze-dried others, and, lastly, carried out SC-CO_2_ drying. These authors pointed out that oven-dried gels were completely collapsed but SC-CO_2_ drying partially shrank the samples—probably due to the weakening of the polymeric network after the alcoholic exchange, which might have modified gel structure due to solubilization or rearrangement effects.

As can be seen from Table 2, HEEPSs are versatile materials in TE applications. However, most papers focus on the biomedical application of HEEPS-based hydrogels and only few of them on the production of dried porous structures. Therefore, due to their promising properties, drying techniques should be explored to assess the utilization of these exopolysaccharides to produce implantable devices.

## 3. Conclusions

To sum up, exopolysaccharides (both homo- and hetero-), which are easily produced by well-known industrial processes (i.e., fermentation from bacteria, fungi, and algae) are particularly interesting for tissue engineering applications because of their ease of production, biocompatibility, non-cytotoxicity, etc. The main drying techniques have been reviewed in this work, especially regarding HOEPSs. From this analysis, it can be concluded that, among all of the proposed drying techniques, SC-CO_2_-assisted drying is the most effective since it enables the production of porous structures that can meet most of the criteria needed for TE applications. Moreover, it is faster (hours vs. days or weeks for freeze-drying and room pressure drying)—which is essential from an industrial perspective—and it can completely remove toxic cross-linker residues from the polymeric network. Moreover, aerogels can mimic human tissues on the nanoscale, and their nanostructure could be a better host space to nanoadditives (needed to adjust polysaccharides’ biomechanical characteristics) since nanopores could prevent cluster formation. Thus, surface effects of the nanoscale are enhanced. However, EPS-based porous structure characteristics rely currently on the materials’ chemistry and structural backbone, solvent medium, gelation mechanism, etc. However, the correct production of such devices of biomedical interest depends on the optimization of a wide range of different operating parameters. In particular, as far as drying is concerned, even though freeze-drying is the most widespread process for scaffold production, SC-CO_2_ drying successfully overcomes the limitations of both FD and RPD. For this reason, the production of both HOEPS- and HEEPS-based porous structures should be further explored to fully valorize their properties and to use them in tissue engineering and regenerative medicine.

## Figures and Tables

**Table 1:** Drying techniques applied to HOEPSs.

HOEPS	Drying Technique	Advantages	Disadvantages	References
DEX	Freeze-drying	Ease of production; non-cytotoxic	Only macroporous structure; incomplete removal of toxic cross-linkers; non-homogeneous distribution of nanoadditives (clusters)	[41,42,43,44]
PUL	Freeze-drying	Ease of production; good mechanical properties	Week-long production; only macropores (100 μm)	[48]
PUL	SC-CO_2_ drying	Hierarchical morphology; intact nanostructure (50 nm pores); high SSA	Poor mechanical properties	[49]
CUR	Room pressure drying	Osteoclast proliferation; good biocompatibility	Closed and smooth surface	[57]
CUR	Freeze-drying	Good mechanical properties; cell proliferation	No nanostructure; incomplete removal of toxic cross-linkers	[58,59]
BC	Freeze-drying	Open interconnected macropores; cell proliferation and differentiation	Up to week-long process; wide pore size distribution; incomplete removal of cross-linkers; collapsed nanostructure	[64,65]
BC	SC-CO_2_ drying	Hierarchical morphology; regularity on microscale and nanoscale; few hours needed for complete drying; high SSA; open and interconnected structure	Poor mechanical properties; fillers needed	[65,66,67,68]
κ-CAR	Freeze-drying	Tunable mechanical properties; osteogenic proliferation	Absence of a hierarchical morphology; no nanostructure observed	[78,79]
κ-CAR	SC-CO_2_ drying	Complete removal of toxic cross-linkers; high SSA; fast process	Shrinkage occurrence	[80,81,82,83]

**Table 2:** TE applications of HA and GG.

HEEPS	TE Application	References
Hyaluronic acid	Vocal fold	[97]
	Bone and cartilage	[98]
	Cardiovascular	[99]
Gellan gum	Neural	[103]
	Connective tissues	[104]
	Myocardial	[105]
	Bones	[106]

## Data Availability

Data are contained within the article.
